# Sub-cellular localization and post-translational modifications of the *Plasmodium yoelii *enolase suggest moonlighting functions

**DOI:** 10.1186/1475-2875-6-45

**Published:** 2007-04-16

**Authors:** Ipsita Pal-Bhowmick, Hardeep K Vora, Gotam K Jarori

**Affiliations:** 1 Department of Biological Sciences, Tata Institute of Fundamental Research, Homi Bhabha Road, Colaba, Mumbai – 400 005, India

## Abstract

**Background:**

Enolase (2-Phospho-D-glycerate hydrolase; EC 4.2.1.11) is one of the glycolytic enzymes, whose levels are highly elevated in malaria parasite infected red blood cells. In several organisms, enolases have been shown to have diverse non glycolytic (moonlighting) biological functions. As functional diversity of a protein would require diverse sub-cellular localization, the possibility of involvement of *Plasmodium *enolase in moonlighting functions was examined by investigating its sub-cellular distribution in the murine malarial parasite, *Plasmodium yoelii*.

**Methods:**

Cellular extracts of *P. yoelii *were fractionated in to soluble (cytosolic) and particulate (membranes, nuclear and cytoskeletal) fractions and were analysed by one and two-dimensional gel electrophoresis. These were probed by Western blotting using antibodies raised against recombinant *Plasmodium falciparum *enolase. Immunofluorescence assay was used for *in situ *localization. Fe^+3 ^based metal affinity chromatography was used to isolate the phospho-proteome fraction from *P. yoelii *extracts.

**Results:**

Apart from the expected presence of enolase in cytosol, this enzyme was also found to be associated with membranes, nuclei and cytoskeletal fractions. Nuclear presence was also confirmed by *in situ *immunofluorescence. Five different post translationally modified isoforms of enolase could be identified, of which at least three were due to the phosphorylation of the native form. *in situ *phosphorylation of enolase was also evident from the presence of enolase in purified phosphor-proteome of *P. yoelli*. Different sub-cellular fractions showed different isoform profiles.

**Conclusion:**

Association of enolase with nuclei, cell membranes and cytoskeletal elements suggests non-glycolytic functions for this enzyme in *P. yoelii*. Sub-cellular fraction specific isoform profiles indicate the importance of post-translational modifications in diverse localization of enolase in *P. yoelii*. Further, it is suggested that post-translational modifications of enolase may govern the recruitment of enolase for non-glycolytic functions.

## Background

Enolase (EC 4.2.1.11) catalyzes the inter-conversion of 2-phosphoglycerate and phosphoenol pyruvate during glycolysis and gluconeogenesis. For many years enolase was regarded as a soluble glycolytic enzyme, exclusively present in cytosol. However, several recent studies have shown that enolase is a multifaceted protein with diverse biological functions and sub-cellular localizations [1,2]. It acts as a plasminogen receptor on the cell surface of certain pathogens [3,4] and has been implicated in nuclear functions in protozoans [5,6], plants [7] and animal cells [8,9]. Enolase is also involved in stress response [10,11] vacuolar fusion processes [12] and molecular chaperoning functions [13,14].

*Plasmodium falciparum *is the causative agent for the most fatal forms of malaria. The asexual blood stages of this parasite, which are responsible for clinical symptoms of the disease, are bereft of functional tricarboxylicacid cycle and solely rely on glycolysis for their energy needs. The infected cells have ~50–100 fold higher glycolytic flux as compared to uninfected red blood cells (RBCs) [15,16]. The levels of some of the glycolytic enzymes are highly elevated and enolase is one such enzyme whose activity levels are ~15–20 fold higher in infected cells [17]. As enolases are known to participate in a host of moonlighting functions, it is likely that it may be recruited for certain other biological functions in the parasite. As involvement of a protein in multiple functions, invariably require its recruitment to different sub-cellular compartments, examination of the sub-cellular localization of enolase in the parasite cells may provide clues to any non-glycolytic functions it may have. Since *Plasmodium yoelii *cells can be obtained easily in large quantities and many of the house keeping proteins are highly homologous with *P. falciparum*, this murine malarial parasite has served as a good model system for human malarial parasite. As enolases from these two organisms are ~90% homologous, antibodies raised against recombinant *P. falciparum *enolase could be used to investigate sub-cellular localization of enolase in *P. yoelli*.

## Materials and methods

### Materials

Recombinant *P. falciparum *enolase (r-Pfen) was purified and polyclonal antibodies were raised in mice as described earlier [18]. Rabbit anti-*P. falciparum *aldolase antibody was a kind gift from Prof. Victor Nussenzweig, Department of Pathology, N.Y. University Medical Centre, New York, USA. All chemicals used were of Analar grade.

### Methods

#### Preparation of *P. yoelii *cells

The mice were infected with *P. yoelii *strain 17XL and the parasitaemia was allowed to reach ~30% level. At this stage, 1–2 ml of blood were collected in equal volume of anticoagulant containing 136 mM glucose, 42 mM citric acid and 75 mM sodium citrate. Blood from five animals was pulled together and RBCs were pelleted at 1500 × g for 5 minutes and washed three times with phosphate buffer saline (PBS) (137 mM NaCl, 2.7 mM KCl, 10 mM Na_2_HPO_4_, 1.8 mM KH_2_PO_4_, pH 7.4). Infected erythrocytes were washed twice in PBS and treated with 0.05% saponin for 10 min at 4°C to release the parasites from the host erythrocyte membrane. The parasite pellet was washed with PBS and stored at -80°C. For the preparation of merozoites, the following procedure was adopted. *P. yoelii *infected blood containing enough mature, segmented schizont-containing erythrocytes was collected and washed with PBS. It was repeatedly passed through a 25-gauge needle to release merozoites. The suspension was centrifuged first at 600 × g to pellet out cellular debris. The supernatant was centrifuged at 8,000 × g for 10 minutes to pellet down the merozoites and washed twice with PBS. Collected merozoites were kept at -80°C till further use.

#### Sub-cellular fractionation

The differential detergent fractionation (DDF) method was used to sequentially solubilize various sub-cellular components [19]. Parasite cell pellet (~10^6 ^cells) was suspended in ice cold Buffer-A (300 mM sucrose, 100 mM NaCl, 3 mM MgCl_2_, 5 mM EDTA, 2 mM PMSF, ROCHE incomplete cocktail protease inhibitor in 10 mM PIPES, pH 7.2) containing 0.02% digitonin for 10 minutes and centrifuged at 1,000 × g. The supernatant is the cytosolic fraction. The pellet was then extracted with ice-cold 1.0 % (v/v) Triton x-100 in buffer-A for 30 minutes and centrifuged at 5,000 × g for 30 minutes. Supernatant obtained represents the solubilized membrane fraction. The resultant pellet was suspended in Buffer-A containing 0.5% deoxycholate, 1.0% Tween-40 and homogenized in a Teflon homogenizer (five strokes) and centrifuged at 7,000 × g for 10 minutes. The solubilized component was the nuclear fraction. Finally, the detergent resistant pellet (cytoskeletal fraction) was dissolved in 5% (w/v) SDS in 10 mM sodium phosphate pH 7.4.

Proteins from all the solubilized fractions were precipitated using trichloroacetic acid (TCA)-acetone. TCA, ice cold acetone and aqueous extracts were mixed in a ratio of 1:8:1 and kept at -20°C for one hour and centrifuged at 11,000 × g for 15 min at 4°C. Supernatant was discarded and the pellet was extensively washed with acetone, air-dried and stored at -80°C till further use.

#### Two-dimensional gel electrophoresis (2DIGE)

2DIGE was performed as described in Bio-Rad manual. For isoelectric focusing, 1 mg of TCA-acetone powder from different fractions was dissolved in 200 μl of 2 % CHAPS, 7 M urea, 2 M thiourea, 50 mM DTT (freshly added), 0.2% IPG buffer, 0.002% bromophenol blue (rehydration buffer) and IPG strips (pH 4–7; size 11 cms, Bio Rad) were passively rehydrated for 12–14 hrs. Typically, focusing was done for 60,000–70,000 V hrs. Second dimension separation was on a 12 % SDS-PAGE gel and enolase isoforms were visualized by Western blotting. The pI values were determined by taking the IPG strip length as 11.2 cms and pH gradient of three units (pH 4–7). A minimum of two independently prepared sub-cellular fractions were analysed for pI determination. From sample to sample the relative pattern of various spots remained unchanged. However, the absolute values of pI varied within a range of ± 0.1 pH units.

#### Indirect immunofluorescence assay

Immunofluorescence assay (IFA) was performed as described earlier [18] using mouse anti-*P. falciparum *enolase antibodies (1:100) and Alexa Fluor 488-conjugated anti-mouse IgG (Molecular Probes Inc.). Parasite nuclei were stained with DAPI (4',6-diamidino-2-phenylindole) at a final concentration of 1 μg·ml^-1^. Slides were examined using an Ultima Confocal System from the Meridian Instruments.

#### Isolation of *P. yoelii *phosphoproteome

For the isolation of phosphoproteome, *P. yeolii *cells were suspended in the lysis buffer (8 M urea, 50 mM Tris pH 5.5, 150 mM NaCl, 1 μM each of leupeptin and pepstatin-A, 1 mM PMSF and with the addition of complete mini, protease inhibitor cocktails EDTA-free, catalog no. 1 836 17, Roche Diagnostics, Germany) (1 ml/gm wet weight of parasite pellet) and subjected to constant mixing for one hour at 4°C. The extract was centrifuged at 20,000 × g for 30 minutes to obtain clear supernatant.

An iron affinity resin was prepared by mixing freshly made 100 mM FeCl_3 _with Affinity Prosep-chelating resin (iminodiaceticacid-agarose) (Millipore) and the excess FeCl_3 _was removed by extensive washing with lysis buffer. For capturing phosphoproteome, the Fe^+3^- beads were mixed with the clear supernatant and allowed to bind for one hour at 4°C. The slurry was poured in a column and lysis buffer was passed till the flow through was free from the proteins. The bound proteome fraction was eluted using EDTA in buffer.

## Results

### Enolase is present in soluble and particulate fractions of *P. yoelii*

**Figure 1:**
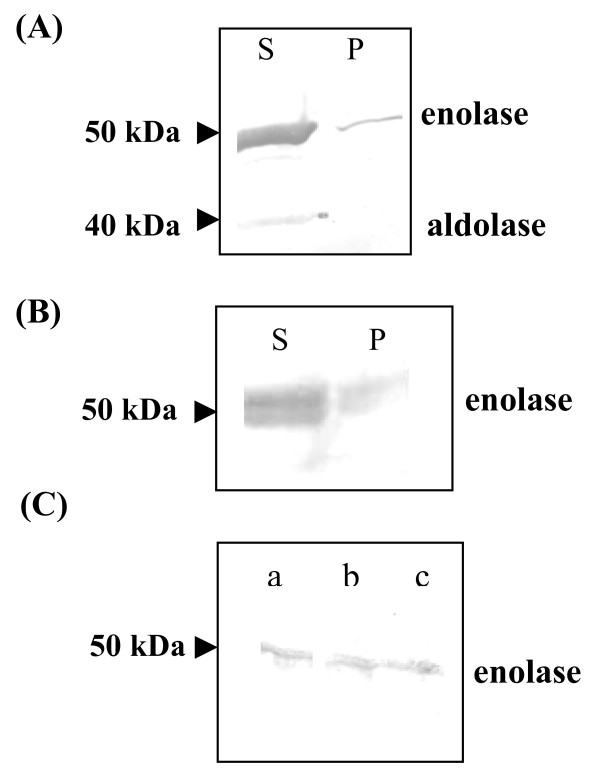
(A) Western blot analysis of soluble (S) and particulate (P) fractions of *P. yoelii *cell extract. The proteins from the two fractions were analysed on a 12% SDS-PAGE and the blot was probed for the presence of enolase (~50 kDa) and aldolase (~40 kDa). (B) Western blot of soluble (S) and particulate (P) fractions of *P. yoelii *merozoite extract. (C) Western blot for the detection of enolase (Pfen) associated with the (a) membrane fraction, (b) nuclear fraction and (c) cytoskeletal fraction. The proteins in the samples were analysed on a 12% SDS-PAGE and the blots were probed using r-Pfen antibodies for the presence of enolase (~50 kDa).

The parasite saponin pellet having about 10^6 ^cells was lysed by freeze-thaw followed by sonication and centrifuged at 14,000 × g for 30 minutes to obtain soluble (supernatant) and particulate (pellet) fractions. The proteins were separated by SDS/PAGE (12% gel) and Western blotting was performed as described earlier [18]. Results are presented in Figure 1A. Enolase (~50 kDa band) was found to be present in both (soluble and particulate) fractions. As expected, most of the enolase was present in cytosol (~85–90%) where it is required for glycolytic function. However, ~10–15% enolase was associated with particulate fraction. As most of the enolase is located in cytosol, there is a possibility of contaminating the particulate fraction with unbroken cells. To eliminate this possibility, the blot was also examined for the presence of another glycolytic enzyme, aldolase (using anti-*P. falciparum *aldolase antibodies). As shown in Figure 1A, aldolase (~40 kDa band) was present only in the soluble fraction and was completely absent from the particulate fraction, suggesting that the pellet fraction did not have any contamination from the cytosolic fraction. Similarly, an extract prepared from the purified merozoites (an extra erythrocytic stage), also showed the presence of enolase in soluble as well as particulate fraction (Figure 1B).

Since the particulate fraction consisted of a variety of sub-cellular components (cellular membranes, nuclei and cytoskeletal elements), differential detergent fractionation (DDF) method was used to selectively solubilize proteins from various organelles [19]. 100 μg acetone powder derived from solubilzed membranes, nuclei and cytoskeletal elements was analysed by SDS/PAGE. Results presented in Figure 1C, showed association of enolase with all the three sub-cellular fractions.

### Immunofluorescence assay (IFA) showed nuclear presence of enolase in *P. yoelii*

**Figure 2:**
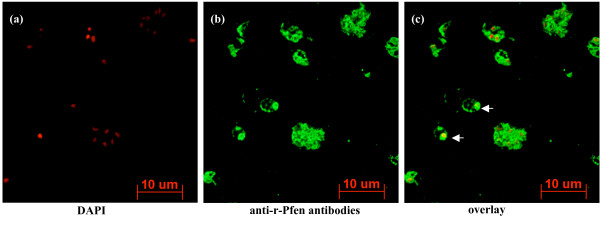
Confocal microscopic study of enolase localization in *P. yoelii *infected mouse red blood cells. Nuclei were stained with DAPI (1 μg·ml^-1^) and the presence of enolase was detected by anti-enolase (anti-r-Pfen) antibodies followed by secondary anti-mouse IgG labeled with alexa fluor 488. (a) DAPI image, (b) mouse anti-r-Pfen antibody and (c) merged image of (a) and (b). Nuclear localization of enolase is marked with arrows.

A smear of infected mouse red blood cells was stained with DAPI to locate the nuclei of parasite cells and with anti-recombinant *P. falciparum *enolase (anti-r-Pfen) antibodies for localization of enolase (Figure 2). An overlay of the two images clearly showed the presence of enolase in the nucleus (shown with arrows). In this smear, it was observed that the presence of enolase is more prominent in early intra-erythrocytic stages (ring and trophozoite) as compared to late multi-nuclear schizont stages. The high specificity of anti-r-Pfen antibodies for parasite enolase is evident from the fact that none of the uninfected RBCs are stained with anti-r-Pfen antibodies in this smear.

### Different isoforms of enolase are associated with various sub-cellular fractions

**Figure 3:**
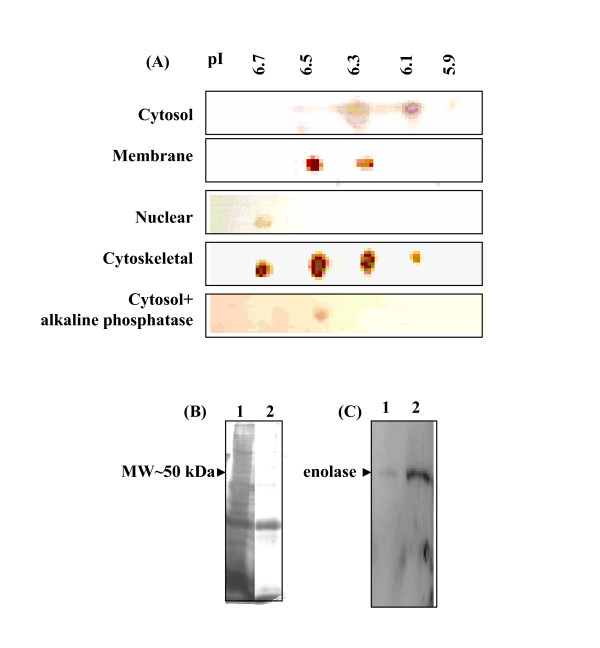
(A) Western blot showing different isoforms of enolase associated with different sub-cellular fractions of *P. yoelii*. 0.8–0.9 mg of acetone powder prepared from cytosol, nuclei, membranes or cytoskeletal components were analysed by two dimensional gel electrophoresis (2DIGE) and transferred to a nitrocellulose membrane. Blots were probed with anti-r-Pfen antibodies. (B) Detection of phosphorylated enolase in *P. yoelii *cell extract. Fe^+3^-iminodiaceticacidagarose beads were used to purify phospho-proteome. Whole cell extract (lane 1) and purified phosphoprotome (lane 2) were analysed on 12% SDS-PAGE and gel was silver stained. (C) Western blot of gel in (B) probed with anti-r-Pfen antibodies.

Using the differential detergent solubilization (DDF) method, the acetone powder was prepared from cytosol, membrane, nuclear and cytoskeletal fractions (full protein complement). These fractions were analysed by 2-dimensional gel electrophoresis (2-DIGE) and probed with anti-r-Pfen antibodies to identify if there are multiple isoforms of enolase in *P. yoelii*. About 0.8–0.9 mg of acetone powder was used for each analysis. Western blots of 2-DIGE from four different sub-cellular fractions are presented in Figure 3A. At least five different isoforms of enolase with pI~5.9, 6.1, 6.3, 6.5 and 6.7 are observed. Different sub-cellular fractions showed differences in the type of isoforms present and their relative abundances. The cytosolic fraction had four different spots (pI~5.9, 6.1, 6.3 & 6.5) with the isoform at pI~6.3 being the most abundant and the form with pI~5.9 being the least. In this fraction, an extra spot at pI~6.5 (and possibly also at 6.3) was observed which has greater electrophoretic mobility. Membrane fraction showed two forms with pI~6.3 and 6.5, whereas the nuclear fraction had a single isoform with a pI~6.7. The cytoskeletal fraction had four isoforms (pI~6.1, 6.3, 6.5 and 6.7), present in almost equal abundance.

In order to examine whether any of these isoforms have arisen due to phosphorylation of native enolase, cytosolic fraction was treated with alkaline phosphatase and analysed. This sample gave a single spot at pI~6.5, suggesting that indeed the isoforms with pI~5.9, 6.1 and 6.3 arose due to phosphorylation of native enolase. Further evidence for *in vivo *phosphorylation of enolase was obtained by pulling out the phosphoproteome fraction from *P. yoelli *cellular extract using Fe^+3^-immobilized metal ion chromatography (IMAC) [20]. If the enolase was phosphorylated inside the cell, IMAC eluted fraction would contain enolase. Figure 3B shows a silver stained gel of *P. yoelii *whole cell extract (lane 1) and EDTA eluted phopho-proteome from Fe^+3^-beads (lane 2). A blot of this gel was probed with anti-r-Pfen antibodies (Figure 3C). The results showed high enrichment of enolase in metal affinity column eluted sample, supporting the view that a major fraction of the enolase is indeed phosphorylated inside the cell.

## Discussion

In this study, two different experimental approaches were employed, namely (i) biochemical sub-cellular fractionation followed by Western blot analysis and (ii) *in situ *location by immunofluorescence to investigate the sub-cellular distribution of enolase in *P. yoelii*. An interesting early result in this study was the observation of the presence of enolase in the particulate fraction (Figure 1). Since glycolysis occurs in cytosol and intra-erythrocytic stages of *Plasmodium *are known to have high glycolytic flux [15,16], it was expected that major fraction of enolase will be present in cytosol. Results presented in Figure 1A suggest that ~85–90% of enolase is cytosolic and ~10–15% is associated with particulate fraction. In cases where a protein is highly abundant in a specific sub-cellular compartment (cytosol here), one needs to demonstrate that the particulate fraction is not contaminated with cytosol and/or unbroken cells. The following evidence supports the view that in case of *P. yoelii*, enolase is indeed associated with various components of particulate fraction:

(i) The presence of aldolase (another glycolytic enzyme) in soluble and particulate fractions was examined. Results presented in Figure 1A showed that the aldolase was present in cytosol and completely absent (or undetectable) in particulate fraction, indicating that particulate fraction is not contaminated with cytosol or unbroken cells;

(ii) in differential detergent solubilization of cellular membrane, nuclei and cytoskeletal elements, enolase was present in all three fractions (Figure 1B). It is highly unlikely that soluble enolase will remain associated in this multi-step detergent solubilization protocol;

(iii) profiles of various enolase isoforms (in terms of their pI and relative abundances) associated with each of the sub-cellular (cytosolic, membrane, nuclear and cytoskeletal) fractions is rather unique (Figure 3A). If the presence of enolase in these fractions was due to cytosolic contamination, one would expect to observe similar profile for all the fractions; (iv) nuclear presence of enolase is also evident from immuno fluorescence assay (Figure 2). All the evidence presented here showed diverse localization of enolase where it may have different physiological (moonlighting) functions. Immunofluorescence assay performed for *in situ *nuclear localization of enolase showed the presence of variable amounts of enolase in different stages of the parasite. For instance, ring stage parasite has lot more nuclear enolase as compared to late multi-nuclear schizont stage (Figure 2). Similar observations have been reported for *T. gondii *[5] and *E. tenella *[6]. Such observations of the nuclear presence of enolase, has lead to the suggestion that it may play a role in gene expression regulation. In this context, recent report about direct interaction of enolase with H3- histone, nucleosome assembly protein and indirect interaction to many other nuclear proteins (including histone acetylase like enzymes) in *P. falciparum *assumes significance [21].

*P. yoelii *has a single gene for enolase. Observation of five different isoforms (pI~5.9, 6.1, 6.3, 6.5 and 6.7) suggests that four of these variants arose due to post-translational modifications. Results presented here showed that forms with pI~5.9, 6.1 and 6.3 arose due to multiple phosphorylations. It is interesting to note that none of these phosphorylated forms move to the nucleus. Phosphorylation of enolase has been widely reported in bacteria [22], plants [23,24] and animals [25,26]. However, the physiological significance of these modifications is not understood. Enolases are highly conserved proteins across the species and do not possess any signal sequences for specific sub-cellular localization. The observation (Figure 3) of sub-cellular fraction specific isoform profile would suggest that post-translational modifications may regulate sub-cellular localization of enolase. The nuclear localization signal (NLS) in a protein is usually a stretch of basic aminoacids [27] and no such sequence is present in the enolase. It is interesting to note that the form which gets translocated to the *Plasmodium *nucleus has the most basic pI (pI~6.7) among all the isoforms.

## Conclusion

In summary, in this paper it was demonstrated that in *P. yoelii*, a small fraction of enolase is associated with cellular membranes, cytoskeletal and nuclear fractions where it is likely to have diverse moonlighting functions. Further, the enolase undergoes several posttranslational modifications, three of which are due to protein phosphorylations. The acidic forms generated due to *in situ *phosphorylations, are excluded from nuclear localization. Variable amounts of enolase detected in nucleus at different life cycle stages of the parasite, suggests nuclear functions for enolase. Implicit in the diverse localization of enolase, is the complexity in its biological functions, which would make this protein an interesting drug target for malaria.

## Authors' contributions

IPB prepared the enolase, raised polyclonal antibodies and carried out biochemical fractionations and immuno fluorescence studies. HKV performed IMAC for phosphoproteome purification. GKJ was involved in design and coordination of the study, assisted in drafting the manuscript. All authors have read the manuscript and approved the final version submitted.
